# RICTOR Affects Melanoma Tumorigenesis and Its Resistance to Targeted Therapy

**DOI:** 10.3390/biomedicines9101498

**Published:** 2021-10-19

**Authors:** Ahlem Jebali, Maxime Battistella, Céleste Lebbé, Nicolas Dumaz

**Affiliations:** 1 INSERM, U976, Team 1, Human Immunology Pathophysiology & Immunotherapy (HIPI), F-75010 Paris, France; ahlem-jebali@hotmail.fr (A.J.); maxime.battistella@aphp.fr (M.B.); celeste.lebbe@aphp.fr (C.L.); 2 Institut de Recherche Saint Louis (IRSL), Université de Paris, F-75010 Paris, France; 3 Pathology Department, Saint-Louis Hospital, AP-HP, F-75010 Paris, France; 4 Dermatology Department, Saint-Louis Hospital, AP-HP, F-75010 Paris, France

**Keywords:** melanoma, BRAF, BRAF inhibitors, mTOR, mTOR inhibitors, mTORC2, resistance

## Abstract

The network defined by phosphatidylinositol-3-kinase (PI3K), AKT, and mammalian target of rapamycin (mTOR) plays a major role in melanoma oncogenesis and has been implicated in BRAF inhibitor resistance. The central role of RICTOR (rapamycin-insensitive companion of mTOR) in this pathway has only recently begun to be unraveled. In the present study, we assessed the role of mTORC2/RICTOR in BRAF-mutated melanomas and their resistance to BRAF inhibition. We showed that RICTOR was significantly overexpressed in melanoma and associated with bad prognoses. RICTOR overexpression stimulated melanoma-initiating cells (MICs) with ‘stemness’ properties. We also showed that RICTOR contributed to melanoma resistance to BRAF inhibitors and rendered the cells very sensitive to mTORC2 inhibition. We highlighted a connection between mTORC2/RICTOR and STAT3 in resistant cells and revealed an interaction between RAS and RICTOR in resistant melanoma, which, when disrupted, impeded the proliferation of resistant cells. Therefore, as a key signaling node, RICTOR contributes to BRAF-dependent melanoma development and resistance to therapy and, as such, is a valuable therapeutic target in melanoma.

## 1. Introduction

Malignant melanoma, a tumor arising from melanocytes, is the most aggressive form of skin cancer with a high tendency to rapidly metastasize. Despite being a rare type of skin cancer, it is responsible for >70% of skin cancer-related deaths. Fortunately, recent advances in melanoma research have established the molecular oncogenic events that contribute to melanoma initiation and progression, allowing the development of targeted therapies. The discovery of BRAF mutations in 50–60% of primary melanoma has led to the development of BRAF inhibitors (vemurafenib, dabrafenib, and encorafenib), as well as MEK inhibitors (cobimetinib, trametinib, and binimetinib), which have improved prognoses and the overall survival in patients with metastatic BRAF-mutant disease. Despite a remarkable early remission and overall improvement of patient outcome on BRAF inhibitor monotherapy and combination BRAF/MEK inhibitor therapy, resistance to RAF/MEK-targeted therapies invariably occurs and nearly all patients relapse within five years of treatment [1,2,3]. In the majority of progressing melanoma, resistance occurs via the re-activation of MAPK signaling, but a proportion of resistant melanoma relies on the activation of the compensatory phosphoinositide 3-kinase (PI3K) signaling cascade [4]. 

The signaling network of PI3K, AKT, and mammalian target of rapamycin (mTOR) controls most hallmarks of cancer, including cell cycle, survival, metabolism, motility, and genomic instability [5]. The central protein of this pathway is the AKT kinase, which is activated by the PDK1 kinase and the mTORC2 complex [6]. The PI3K signaling pathway is regulated by the PTEN phosphatase, which inhibits its activation. This pathway is very often activated in melanoma by activating mutations of the oncogene NRAS, loss of expression or function of the tumor suppressor gene PTEN, and activation of receptor tyrosine kinases (RTKs) [7]. The activation of this pathway plays a major role in melanoma oncogenesis, cooperating with BRAF mutations to transform melanocytes [8]. Aberrant signaling in the PI3K/AKT pathway has also been implicated in BRAF inhibitor resistance [4]. PTEN loss, which leads to increased PI3K pathway activity, is common in patients treated with the BRAF inhibitor vemurafenib [9], and decreased PTEN expression has been identified as a predictor of decreased progression-free survival (PFS) in patients treated with the drug [10]. When comparing treatment-naïve and dabrafenib- or vemurafenib-treated melanoma patients, several studies have identified PI3K-AKT activation downstream of the platelet-derived growth factor receptor β (PDGFRβ) or the insulin-like growth factor 1 receptor (IGF1R) as a mechanism of therapy resistance [11,12,13]. Similarly, increases in RTK-ligand levels, through autocrine tumor-cell production or paracrine contribution from tumor stroma, confer resistance through activation of the PI3K pathway [14,15]. 

These observations have led to mitogen-activated protein kinase (MAPK) and PI3K dual inhibition strategies to delay resistance in melanoma [16]. Preclinical data have demonstrated the superior antitumor activity of a combination of MAPK and PI3K pathway inhibitors in BRAF-mutant cell lines. Moreover, melanoma cells resistant to BRAF inhibitors have a MEK-independent survival driver that can be blocked by inhibitors of the PI3K pathway [17]. However, the combination of the pan-class 1 P13K inhibitor BKM120 with vemurafenib in both BRAF inhibitor-naïve and -resistant patients with advanced melanoma was poorly tolerated and did not warrant further study [18,19]. Other studies combining MEK inhibitors with PI3K or AKT inhibitors have demonstrated significant toxicity [20,21]. To identify an optimal approach combining inhibition of both the MAPK and PI3K pathways with an acceptable toxicity profile in melanoma, it is essential to better understand the complexity of the PI3K pathway in these cells. We recently highlighted a new alteration of the PI3K pathway in melanoma, demonstrating that the RICTOR locus, which codes for the scaffolding protein of the mTORC2 complex, is frequently amplified in melanomas [22]. Furthermore, the overexpression of RICTOR in melanocytes transformed by the NRAS oncogene stimulates their clonogenicity [22]. These results show that RICTOR plays a central role in the regulation of the PI3K pathway in melanocytes and that its deregulation could be involved in the transformation of melanocytes.

In the present study, we assessed the role of mTORC2/RICTOR in BRAF-mutated melanoma and showed that RICTOR stimulated melanoma-initiating cells (MICs) with ‘stemness’ properties. We showed that mTORC2/RICTOR is an important therapeutic target in BRAF-mutant melanoma, particularly in those resistant to targeted therapy where RICTOR interacts with RAS.

## 2. Materials and Methods

### 2.1. Cell Culture 

Normal Human Epidermal Melanocytes (NHEMs; Promo Cell, Heidelberg, Germany) were cultured in KBM-Gold medium containing 0.5% serum and SCF (Lonza, Bazel, Switzerland). A375, Colo829, and SkMel5 melanoma cell lines were cultured in RPMI 1640 or DMEM (Invitrogen, Cergy Pontoise, France) containing 10% (*v*/*v*) fetal calf serum (FCS; Perbio, Bredières, France), L-glutamin (2 mM; Invitrogen, Cergy Pontoise, France) and antibiotics (100 U/mL of penicillin and 1000 μg/mL of streptomycin; Invitrogen, Cergy Pontoise, France). A375R1 and R2 were derived from A375 by a culture with increasing doses of vemurafenib up to 10 μM. For clonogenic assays, cells were seeded at low density and treated three times a week with inhibitors or DMSO. Cells were fixed and stained after 2 weeks with 0.5% (*v*/*v*) crystal violet + 20% methanol, and colonies were counted. For spheroid culture, 2000 cells were seeded in ultra-low-attachment 6-well plates (Sigma Aldrich Chimie, Saint-Quentin-Fallavier, France) in DMEM/F12 medium supplemented with 1X B27 (Invitrogen, Cergy Pontoise, France), 10 ng/mL of basic fibroblast growth factor (Peprotech, Neuilly-Sur-Seine, France), 20 ng/mL of epidermal growth factor (Peprotech, Neuilly-Sur-Seine, France), and 5 μg/mL of insulin (Sigma Aldrich Chimie, Saint-Quentin-Fallavier, France). The identity of the cell lines used in this study was confirmed by NGS.

### 2.2. Plasmids and Reagents 

Vemurafenib (BRAFV600E inhibitor), OSI-027 (mTOR inhibitor), and SH4-54 (dual STAT 3/5 inhibitor) were obtained from Seleckhem (Houston, TX, USA). JR-AB2-011 (mTORC2 inhibitor) was from MedChemExpress (Monmouth Junction, NJ, USA). Myc epitope-tagged RICTOR was previously described [22]. For stable RICTOR expression, melanoma cells were transfected with JetPEI (Polyplus-transfection, Illkirch, France) according to the manufacturer’s instructions and selected with Blasticidin (10 μg/mL; Gibco, Carsbad, CA, USA). Selected cells were either fixed and stained with 0.5% (*v*/*v*) crystal violet + 20% methanol or collected and grown up for further analysis.

### 2.3. RNA Purification and Quantitative RT-PCR 

Total RNA was extracted from cells cultured as spheroids using Nucleospin RNA kit (MACHEREY-NAGEL GmbH & Co., KG, Duren, Germany). Reverse transcription was performed with the ThermoScript RT–PCR System (Thermo Fisher Scientific, Rockford, IL, USA). Primers were specifically designed for each transcript using Primer Express 3.0 software (DeNovo Software, Glendale, CA, USA). Transcript levels were measured by qRT–PCR using SYBR green master on a 7300 RT–PCR system (Applied Biosystems, Foster City, CA, USA). Transcript levels were normalized with the transcripts from GAPDH. 

### 2.4. Western Blotting and Antibodies

Melanoma cells were lysed using RIPA buffer supplemented with a proteinase inhibitor cocktail. Protein concentration was quantified by a Bradford protein assay (Protein Assay Kit, BioRad, Hercules, CA, USA). Whole-cell lysates were resolved by SDS–polyacrylamide gel electrophoresis and transferred on nitrocellulose membranes. Membranes were probed with the following primary antibodies: Myc-Tag, RICTOR, ACTIN, p-AKT (S473), AKT, p-ERK, ERK, pSTAT3 (Y705), STAT3, SOX10, AXL (Cell Signaling Technology, Danvers, MA, USA), and MITF (OriGene Technologies Inc, Rockville, MD, USA). Proteins were revealed with a SuperSignal^®^ West Pico Chemiluminescent Substrate (Thermo Scientic, Rockford, IL, USA) on an ImageQuant imaging system and quantified using Image J software (NIH). The quantification of Western blots is presented in Figure S8.

### 2.5. Human Melanoma Samples 

From 2015 to 2018, paraffin-embedded (FFPE) tissue specimens were collected from the Dermatology Department of Saint Louis Hospital, Paris, France for 5 patients with metastatic melanoma before treatment with a combination of dabrafenib plus trametinib and after resistance occurred. All patients were included in the Melbase cohort (ClinicalTrials.gov Identifier: NCT02828202) and gave informed written consent. The research was approved by the Ethics Committee of Saint Louis Hospital. The clinical data of the 5 patients are presented in Table S1.

### 2.6. Proximity Ligation Assay 

Cells grown on 12-well culture slides were fixed in 4% PFA and permeabilized with 0.5% Triton X100 before being subjected to in situ PLA using the Duolink Detection kit (Sigma Aldrich Chimie, Saint-Quentin-Fallavier, France) according to the manufacturer’s instructions. For formalin-fixed paraffin-embedded (FFPE) tissue sections, five micrometer-thick sections were dewaxed in xylene and rehydrated using decreasing concentrations of alcohol. After antigen retrieval in 10 mM of citrate sodium buffer (pH 6) for 15 min at 95 °C, sections were subjected to PLA using the Duolink Detection kit (Merck) according to the manufacturer’s instructions. The samples were blocked, incubated with antibodies directed against RICTOR (A304; Bethyl Laboratories, Montgomery, TX, USA) and RAS (F132; Santa Cruz Technologies, Heidelberg, Germany) or mTOR (Cell Signaling Technology, Danvers, MA, USA), and thereafter incubated with PLA probes, which are secondary antibodies conjugated to oligonucleotides. Circularization and ligation of the oligonucleotides were followed by an amplification step. The products were detected by a complementary fluorescently labeled probe. Protein complexes were visualized with a fluorescent microscope. 

### 2.7. Statistical Analysis 

Survival outcome analysis of using the TCGA PanCancer Atlas-Skin Cutaneous Melanoma dataset was performed according to Liu and colleagues’ guidelines [23]. GSE dataset analyses were performed using Prism 6 (GraphPad Software Inc., La Jolla, CA, USA), and unpaired *t*-tests were used to evaluate differences between two groups. Data are presented as the mean values ± s.d with *p*-values indicated as * *p* < 0.05, ** *p* < 0.01. 

## 3. Results

### 3.1. RICTOR Expression in Human Melanocytes and Melanoma Cells and Tissues

**Figure 1:**
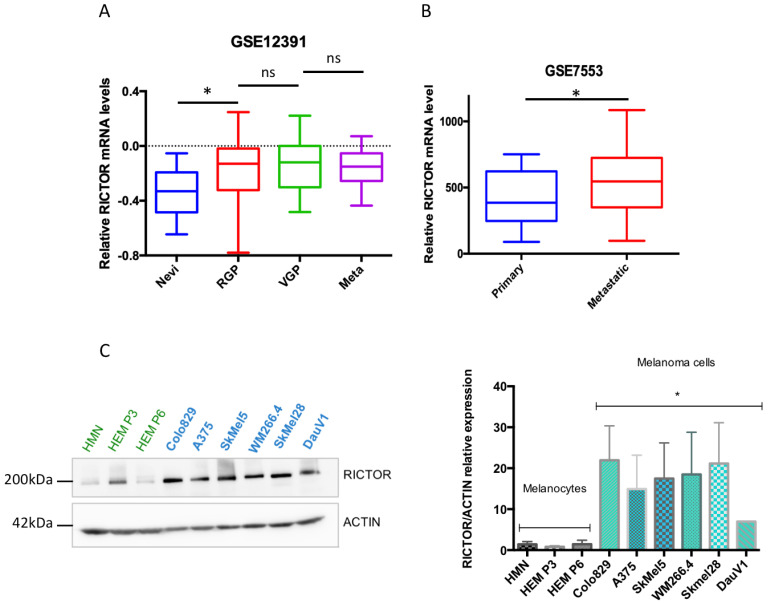
RICTOR expression in human melanocytes and melanoma cells and tissues. (**A**) Bio-informatic analysis of RICTOR expression at the mRNA level in human nevi (Nevi; *n* = 31), radial growth phase (RGP; *n* = 11), vertical growth phase (VGP; *n* = 25), and metastasis melanoma (Meta; *n* = 5). GSE12391 raw data were compared using the Mann–Whitney test. * *p* < 0.05. (**B**) Bio-informatic analysis of RICTOR expression at the mRNA level in human primary melanoma (Primary; *n* = 14) and metastasis melanoma (Metastatic; *n* = 40). GSE7553 raw data were compared using the Mann–Whitney test. * *p* < 0.05. (**C**) RICTOR protein expression was assessed by Western blotting in melanocytes (three independent donors, in green) and six melanoma cell lines mutated on BRAF (in blue). β-Actin served as a loading control. RICTOR protein expression was quantified on three independent experiments and normalized with β-Actin expression. The graph represents the means ± s.d. RICTOR expression in the three melanocytes cell lines was compared to its expression in the six melanoma cell lines using the unpaired Student’s *t*-test: * *p* = 0.0022.

We have previously shown that RICTOR is responsible for an important negative feedback in the PI3K pathway in melanocytes, restricting their proliferation. Furthermore, in a series of 43 melanoma short-term cultures, we showed that the RICTOR locus was amplified in 19 out of 43 melanoma cell lines (44%) and that amplification was independent of the BRAF and NRAS mutation status [22]. To address the role of RICTOR in melanoma, we first analyzed the expression of RICTOR in previously published gene expression datasets of patient-derived melanoma samples. Analysis of the GSE12391 melanoma data demonstrated that RICTOR was significantly overexpressed in patient-derived melanoma samples compared to nevi samples (*p* = 0.019; Figure 1A), but was not significantly increased in metastatic melanoma (*n* = 5) compared to primary melanoma. However, bioinformatics analysis of another set of data containing more metastatic samples (GSE7553) showed that RICTOR was significantly overexpressed in metastatic melanoma (*n* = 40) compared to primary melanoma (*p* = 0.044; Figure 1B). This agrees with a previous small study that identified an increased expression of RICTOR in advanced melanoma stages including liver metastases, suggesting a role of RICTOR in melanoma progression and metastasis [24]. We then analyzed the relevance of RICTOR expression on overall survival in the TCGA cohort of melanoma patients. We found that a high expression of RICTOR was associated with a shorter overall survival (*p* = 0.023; Supplementary Figure S1A). Interestingly, a high mTOR expression was not associated with a shorter overall survival, and a high expression of RAPTOR, the scaffolding protein of the mTORC1 complex, was associated with good prognosis (*p* = 0.05; Supplementary Figure S1B). These data highlight a more important role of mTORC2/RICTOR than mTORC1/RAPTOR in melanoma development and progression. These findings are consistent with a previous report, which detected RICTOR by immunohistochemistry and associated a high RICTOR protein level with a shorter overall survival in melanoma [25]. Interestingly, analyzing the gene expression associated with RICTOR expression in the TCGA database, we found that an increased expression of RICTOR was correlated with an increased expression of BRAF (Supplementary Figure S1C). We therefore concentrated our ex vivo studies on BRAF-mutated melanoma. We first assessed the RICTOR expression in melanocytes and BRAF-mutated melanoma by Western blot. We used three independent primary cultures of melanocytes and seven BRAF-mutated melanoma cell lines and found that the RICTOR protein was significantly overexpressed in melanoma cell lines compared to melanocytes, suggesting a correlation between RICTOR expression and BRAF-dependent melanoma development (Figure 1C).

### 3.2. RICTOR Overexpression Promotes Cell Growth in Spheroid Culture

**Figure 2:**
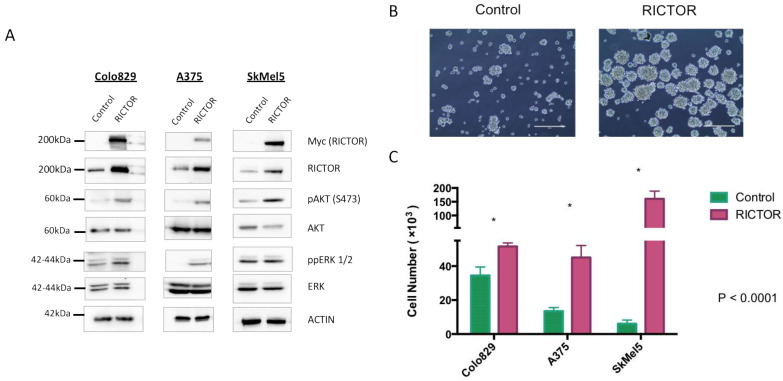
RICTOR overexpression promotes cell growth in spheroid culture (**A**) Colo829, A375, and SkMel5 melanoma cell lines were transfected with an empty vector (Control) or with a vector expressing human RICTOR cDNA (RICTOR). The expression of Myc-tag RICTOR, total RICTOR, phosphorylated AKT and ERK, and total AKT and ERK were assessed by Western blotting. (**B**,**C**) Colo829, A375, and SkMel5 cell lines transfected with an empty vector (Control) or with a vector expressing human RICTOR cDNA (RICTOR) were grown as a spheroid for 7 days and counted. Pictures from SkMel5 cell lines were taken after 7 days; scale bar represents 1000 μm. Graphs represent the mean ± s.d. of an experiment in triplicate (similar results were obtained in three independent experiments). Significance was calculated using the Student’s *t*-test: * *p* < 0.05.

To gain insights into the role of RICTOR in melanoma development, we overexpressed RICTOR in three BRAF-mutated melanoma cell lines (Colo829, A375, and SkMel5). Using Western blot, we first confirmed RICTOR overexpression in the three cell lines and showed that it was associated with an increase in the phosphorylation of AKT on Ser473 (Figure 2A). Interestingly, in one cell line, ERK phosphorylation was very low in the parental cells but increased in cells overexpressing RICTOR probably due to cross-talk and compensation between the PI3K and the MAPK pathways in these cells. To test the biological effect of RICTOR overexpression, we measured its effect on proliferation. As shown in Figure 2B,C, RICTOR overexpression significantly increased melanoma cell growth as spheroids. This increase was due to an increase in the number of spheres, as well as an increase in the size of the spheres (Supplementary Figure S2A). This effect was, however, not seen in cells grown in two dimensions where RICTOR overexpression inhibited proliferation (Figure S2B). 

**Figure 3:**
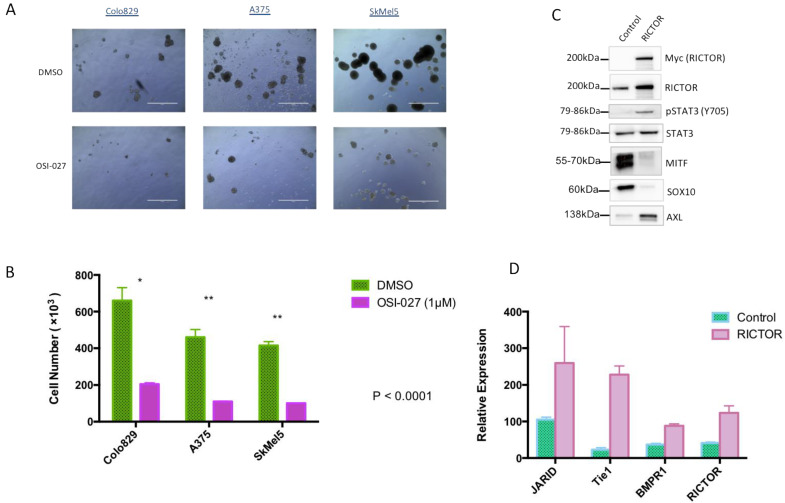
RICTOR-mediated melanoma spheroid growth induces an MIC phenotype (**A**,**B**) Colo829, A375, and SkMel5 cell lines were cultivated under the same condition as Figure 2 and treated with DMSO or an mTOR inhibitor (OSI-027 1 μM) for 10 days and counted (scale bar represents 1000 μm). Graphs represent the mean ± s.d. of an experiment in triplicate (similar results were obtained in three independent experiments). Significance was calculated using the Student’s *t*-test: * *p* < 0.05, ** *p* < 0.01. (**C**) The expressions of Myc-tagged RICTOR, RICTOR, phosphorylated STAT3 (Y705), total STAT3, MITF, SOX10, and AXL were assessed by Western blotting in Colo829 cells transfected with an empty vector (Control) or with a vector expressing human RICTOR cDNA (RICTOR). (**D**) Relative mRNA expressions of the different tumor-initiating cell markers (JARID, Tie1, and BMPR1), as well as RICTOR, were assessed by qPCR and normalized to GAPDH in Colo829 cells transfected with an empty vector (Control) or with a vector expressing human RICTOR cDNA (RICTOR).

To confirm the implication of the mTORC2/RICTOR pathway in spheroid growth, we treated them with a dual mTORC1/mTORC2 inhibitor (OSI-027). After 10 days of treatment, a significant reduction in spheroid growth was seen in cells overexpressing RICTOR compared to control-treated cells (Figure 3A,B). This result demonstrated that the increase in spheroid growth of cells overexpressing RICTOR was due to the increase in mTORC2 activity in these cell lines. The formation of spheres by melanoma cells under nonadherent conditions has been described as characteristic of melanoma-initiating cells (MICs)-less differentiated cancer cells with a higher ability to form tumors [26,27]. Considering the effect of RICTOR/mTORC2 on spheroid growth, we analyzed the expression of ‘stemness’ markers in RICTOR-overexpressing Colo829 cells. We showed that the overexpression of RICTOR was associated with an increase in STAT3 phosphorylation, decreases in MITF and SOX10 expressions, but an increase in AXL expression (Figure 3C). We also evaluated the expression at the mRNA level of three markers of stem cells: JARID, Tie1, and BMPR1, and showed that their expression was increased in cells overexpressing RICTOR (Figure 3D). Altogether, these results strongly supported the acquisition of a MIC phenotype by cells overexpressing RICTOR, which is dependent on mTORC2 activation. To evaluate the role of the STAT pathway in RICTOR-overexpressing cells, we treated the control and the overexpressing cells grown as spheroids with a dual STAT3/STAT5 inhibitor. Although both control and RICTOR cells were sensitive to the STAT inhibitor, the cells overexpressing RICTOR were almost 10 times more sensitive than the control cells were (Supplementary Figure S3). This result demonstrated the importance of the STAT pathway downstream of mTORC2 in RICTOR-overexpressing cells.

### 3.3. RICTOR Overexpression Contributes to Melanoma Targeted Therapy Resistance

**Figure 4:**
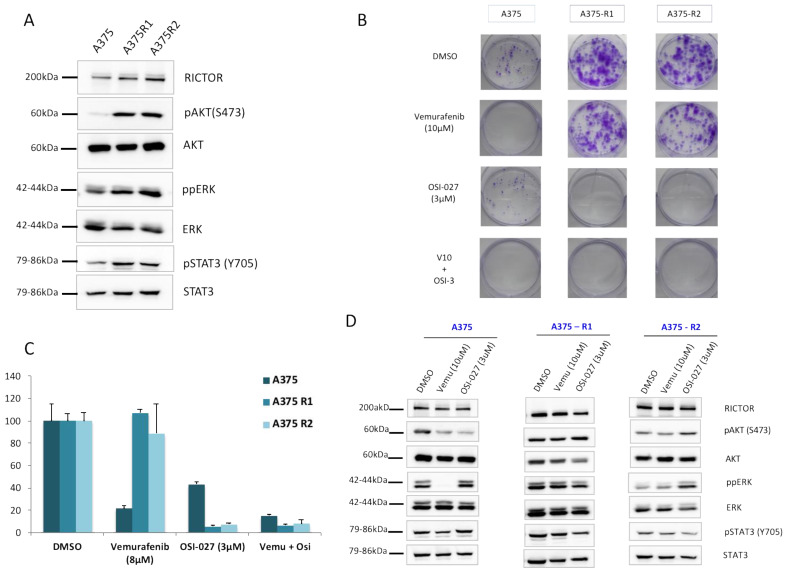
RICTOR overexpression contributes to melanoma-targeted therapy resistance through STAT pathway (**A**) The expression of RICTOR, phosphorylated AKT and ERK, total AKT and ERK, phosphorylated STAT3 (Y705), and total STAT3 were assessed by Western blotting in the A375 melanoma cell line and two independent clones of A375 Vemurafenib resistant to vemurafenib (A375R1 and A375R2). (**B**,**C**) A375 parental and resistant cell lines cells were seeded at low density and treated three times a week with inhibitors or DMSO, Vemurafenib (10 μM), OSI-027 (3 μM), or the combination of both for 10 days. Graphs represent the mean ± s.d. of an experiment in triplicate (similar results were obtained in three independent experiments). (**D**) A375 parental and resistant cell lines cells were treated for 24 h with DMSO, Vemurafenib (10 μM), or OSI-027 (3 μM), and the expressions of RICTOR, phosphorylated AKT and ERK, total AKT and ERK, phosphorylated STAT3 (Y705), and total STAT3 were assessed by Western blotting.

As the increases in STAT3 phosphorylation and AXL expression associated with a decrease in MITF expression have been associated with the resistance of melanoma cells to targeted therapies, we asked whether RICTOR/mTORC2 could play a role in resistance. We first quantified the RICTOR expression in cells, which had been made resistant to BRAF inhibitors. We observed, in two independent pools of resistant cells, an overexpression of RICTOR compared to the parental cell lines (Figure 4A). This was associated with an increase in AKT phosphorylation, as well as an increase in STAT3 phosphorylation in the resistant cells. To confirm the implication of the mTORC2/RICTOR pathway in resistance, we treated the parental and resistant cells with an mTOR inhibitor in a clonogenic assay. As expected, the parental cells were more sensitive to a BRAF inhibitor (vemurafenib) than the mTOR inhibitor (OSI-027) (Figure 4B). However, the cells resistant to the BRAF inhibitor were very sensitive to the mTORC2 inhibition, which inhibited proliferation by more than 90% compared to 55% inhibition in control cells (Figure 4C). Using Western blot, we confirmed that ERK phosphorylation was inhibited by vemurafenib in the parental but not the resistant cells. Interestingly, whereas the mTOR inhibitor induced a reduction in AKT phosphorylation in the parental cell lines, we could not detect a similar decrease in the resistant cells even after 24 h of treatment (Figure 4D). However, AKT phosphorylation was reduced in the parental and resistant cell lines after a treatment of only 1 h, demonstrating a more transient effect of the mTOR inhibitor on AKT activation in resistant versus sensitive cells (Supplementary Figure S4). We detected a decrease in STAT3 phosphorylation in the resistant cells but not in the sensitive ones treated with the mTOR inhibitor, confirming a link between mTORC2/RICTOR and STAT3 in resistant cells (Figure 4D). 

**Figure 5:**
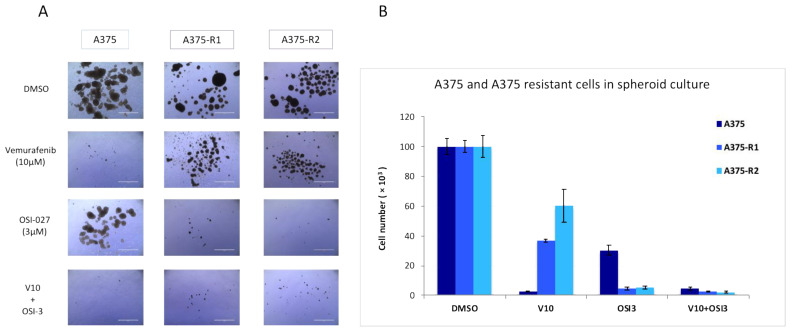
The implication of mTORC2/RICTOR in the resistance of melanoma to targeted therapies in cells grown as spheroids. (**A**,**B**) A375 parental and resistant cell lines were grown as spheroids and treated with DMSO, Vemurafenib (10 μM), OSI-027 (3 μM), or the combination of both for 10 days and counted. Pictures were taken after 10 days of treatment; scale bar represents 1000 μm. Graphs represent the mean ± s.d. of an experiment in triplicate (similar results were obtained in three independent experiments).

To confirm the importance of mTORC2/RICTOR in the resistance of melanoma to targeted therapies in a more physiological setting, we used cells grown as spheroids. We showed in this setting that although the parental cells were more sensitive to a BRAF inhibitor than the mTOR inhibitor, the cells resistant to the BRAF inhibitor were very sensitive to mTORC2 inhibition (Figure 5A,B), highlighting the importance of mTORC2 in resistance to therapy.

### 3.4. RAS–mTORC2 Interaction Is Involved in Melanoma Resistance

**Figure 6:**
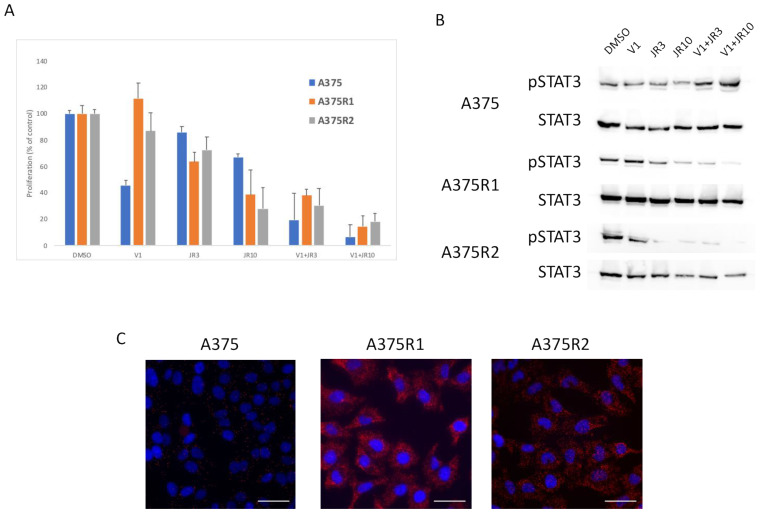
RAS interacts with RICTOR to promote resistance. (**A**) Cells were treated with DMSO, 1 μM of vemurafenib (V1), 3 μM (JR3) or 10 μM (JR10) of JR-AB2-011, or a combination of vemurafenib and JR-AB2-011, and the proliferation was analyzed after 3 days (data are represented as mean +/− SD). Significance was calculated using the Student’s *t*-test and showed that JR10 significantly inhibited proliferation of all 3 cell lines even when combined with V1 (*p* < 0.05). (**B**) A375 melanoma cell line and A375 Vemurafenib-resistant cell lines (A375-R1 and A375-R2) were treated for 24 h with DMSO, Vemurafenib (1 μM), 3 μM (JR3) or 10 μM (JR10) of JR-AB2-011, or a combination of vemurafenib and JR-AB2-011, and the expressions of phosphorylated STAT3 (pSTAT3) and total STAT3 were assessed by Western blotting. (**C**) RAS interactions with RICTOR in A375 parental and resistant cell lines cells detected by in situ PLA. The interactions were visualized as fluorescent red dots. DAPI-stained nuclei (blue). Scale bar, 50 μm.

To confirm the role of mTORC2 in resistance, we used a recently described inhibitor (JR-AB2-011), which specifically inhibits the interaction of RICTOR with mTOR [28]. We treated the parental and resistant cells with JR-AB2-011 alone or in combination with vemurafenib in a proliferation assay. As expected, the parental cells were more sensitive to the BRAF inhibitor (vemurafenib) than the mTORC2 inhibitor (JR-AB2-011) (Figure 6A). However, the cells resistant to the BRAF inhibitor were very sensitive to the mTORC2 inhibition, particularly when combined with vemurafenib, which inhibited proliferation by more than 80% (Figure 6A). To confirm the inhibitory effect of JR-AB2-011 in a more physiological setting, we used cells grown as spheroids. We showed in this setting that the parental cells were more sensitive to a BRAF inhibitor than the mTORC2 inhibitor, but the cells resistant to the BRAF inhibitor were very sensitive to mTORC2 inhibition (Supplementary Figure S5), confirming the importance of mTORC2 in resistance to therapy. Using Western blot, we detected a decrease in STAT3 phosphorylation upon treatment with the mTORC2 inhibitor in the resistant cells but not the sensitive ones, confirming a link between mTORC2 and STAT3 in resistant cells (Figure 6B). To understand the sensitivity of resistant cells to mTORC2 inhibition, we investigated whether RAS directly binds and activates mTORC2 in resistant cells, as recently described in RAS-mutated melanoma [29]. We used proximity ligation assay (PLA), which detects endogenous proteins within 30–40 nM to produce a fluorescent signal. We detected a low PLA signal in the A375 parental cell line but a statistically significant (*p* < 0.05) increase in PLA signal in both resistant lines, demonstrating a strong interaction between RAS and RICTOR in resistant cells (Figure 6C). We confirmed that the treatment of cells with the mTORC2 inhibitor (JR-AB2-011) disrupted mTOR interaction with RICTOR and with RAS (Supplementary Figure S6). 

**Figure 7:**
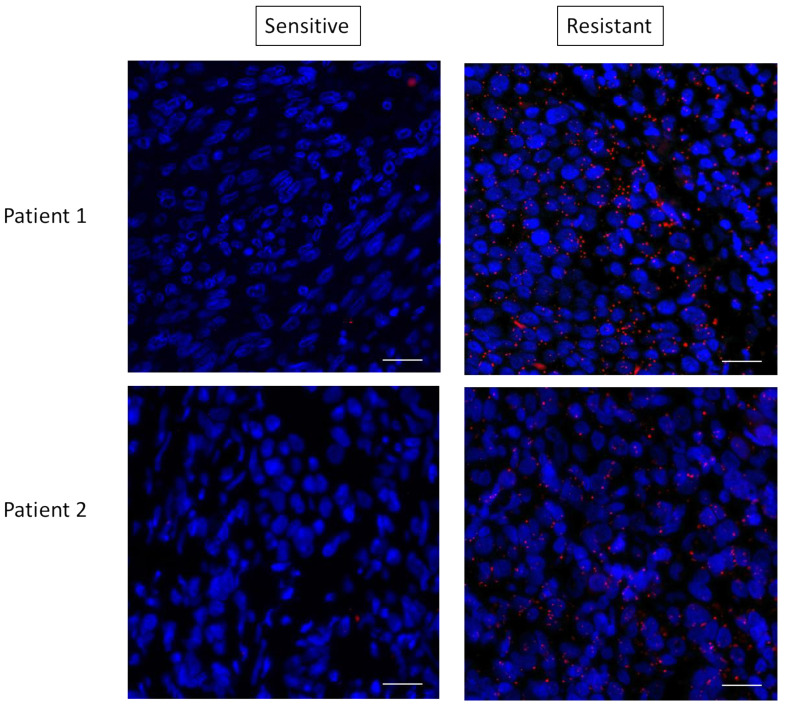
RAS interacts with RICTOR in resistant melanoma. Representative pictures of RAS–RICTOR interactions assessed by in situ PLA (red dots) in paraffin sections of 2 metastatic human melanoma samples before treatment and after resistance occurred. DAPI-stained cell nuclei (blue). Scale bar, 50 μm.

These data suggested that mTORC2 could mediate resistance through interaction with RAS. To determine if this RAS–mTORC2 interaction could be associated with the resistance to targeted therapy in patients, we set out to extrapolate the results we observed ex vivo in human melanoma samples. We tested the RAS–RICTOR interaction using PLA in situ in melanoma sections obtained from five patients before targeted treatments with BRAF and MEK inhibitors and when resistance occurred (Figure 7 and Figure S7). As observed in cultured cells, we found that RAS and RICTOR co-localized in melanoma tumors resistant to therapy. Interestingly, in 4 out of 5 patients, we detected more RAS–mTORC2 interactions in resistant tumors compared to untreated tumors, confirming that this interaction could be involved in melanoma resistance.

## 4. Discussion

Although major improvements for the treatment of melanoma have been made over the past two decades, only a fraction of patients experience long-term benefits from current targeted therapies and immuno-therapies [30]. Identification of new anti-melanoma compounds is therefore essential to effectively treat these tumors. Our study focused on the role of the scaffolding protein RICTOR, a key component of mTORC2 that is required for mTORC2 function. Alterations in RICTOR have been identified in a number of cancer cell types, and its involvement in tumorigenesis and resistance to therapies has recently begun to be unraveled [31]. We have previously shown that RICTOR plays a central role in the regulation of the PI3K pathway in melanocytes and that its deregulation could be involved in the transformation of melanocytes [22]. We have shown that the RICTOR locus is frequently amplified in melanomas and that the overexpression of RICTOR in melanocytes transformed by the NRAS oncogene stimulates their clonogenicity [22]. RICTOR’s deregulation could therefore be involved in the transformation of melanocytes. Data mining performed on The Cancer Genome Atlas (TCGA) Melanoma PanCancer Atlas dataset showed that RICTOR expression is associated with melanoma progression and lower patient survival and might therefore be used as a novel biomarker for prognosis provided it is confirmed in independent studies. To further investigate RICTOR implication in melanoma progression, we assessed whether RICTOR promoted malignant cancer phenotypes in melanoma cells in culture. As RICTOR overexpression in the TCGA dataset was correlated with the overexpression of BRAF, we focused our study on BRAF-mutated melanoma. We showed that RICTOR overexpression promotes cell growth in a spheroid culture in an mTORC2 activity-dependent manner, whereas it inhibited proliferation in cells grown in two dimensions. The difference of the effect of RICTOR overexpression in 2D versus 3D culture highlights the importance of RICTOR in the proliferation and survival of MICs. Interestingly RICTOR overexpression is associated with a decrease in MITF, a key molecular switch that controls the transition between MICs and their differentiated progeny [32]. This was confirmed by the increase in ‘stemness’ markers in RICTOR-overexpressing cells and, in particular, the increase in STAT3 phosphorylation. STAT3, as a point of convergence of many signaling pathways, has been implicated in melanoma progression. The level of phosphorylation of STAT3 is higher in metastasis (particularly the brain and lung) than in cutaneous primary melanomas and is a negative prognostic factor for overall survival in patients who did not develop central nervous system metastasis [33]. Therefore, as with RICTOR, STAT3 alterations are associated with the malignant behavior phenotype in melanoma [34]. As RICTOR overexpression led to an increase in the phosphorylation level of STAT3, it suggests that RICTOR implication in melanoma malignant behavior may be partly linked with STAT3. This is confirmed by the fact that RICTOR-overexpressing melanospheres are almost 10 times more sensitive than the control cells are to the STAT inhibitor. Moreover, STAT3 activation is also an important mechanism of resistance to targeted therapies against mutant BRAF [35]. Our data suggest that the regulation of the STAT pathway by RICTOR could, at least, mediate resistance in BRAF-mutated melanoma cells. Indeed, cells resistant to BRAF inhibitors are very sensitive to mTORC2 inhibition, which induced a decrease in STAT3 phosphorylation, confirming a link between mTORC2/RICTOR and STAT3 in resistant cells. Although acquired activation of PI3K signaling occurs in a subset of resistant melanomas, it remains unclear whether PI3K activation alone is sufficient for the development of BRAF inhibitor resistance. For instance, a baseline expression of phosphorylated AKT or the loss of PTEN is not associated with a response to BRAF inhibition. Complete and partial responses to BRAF inhibition have also been observed in patients with complete loss of PTEN tumor expression and with PIK3CA tumor-associated activating mutations [36,37]. The PI3K pathway likely plays a subtler role in the reorganization of signaling circuits in melanoma cells under the selective pressure of MAPK inhibition. Evidence suggests that, in response to combination BRAF/MEK inhibition, PI3K/AKT activity promotes survival but not proliferation [36]. By allowing the persistence of dormant tumor cells under treatment, activation of the PI3K pathway will have critical implications for the evolution of resistance. Our data suggest that RICTOR overexpression by activating mTORC2 may enable the survival of a dormant subpopulation of MIC. This RICTOR/mTORC2-mediated survival would favor the acquisition of MAPK-reactivating alterations and resistance to targeted therapies. The importance of mTORC2 in resistance could be mediated through its interaction with RAS that is often activated in resistant melanoma by mutation or downstream of RTK [38]. Indeed, we detected the RAS–RICTOR interaction not only in resistant cells but also in biopsies of melanoma patients with resistance to BRAF-inhibition, and showed that disruption of the mTORC2–RAS interaction using JR-AB2-011 impeded the proliferation of resistant cells. 

Micevic et al. previously showed that DNMT3b modulated melanoma development by controlling the level of RICTOR [39], suggesting that mTORC2 signaling was critical for melanoma formation. Our results highlight the importance of RICTOR in melanoma progression, in agreement with Werzowa et al., who previously showed that the silencing of RICTOR reduced melanoma cell line viability and increased apoptosis [40]. These studies have outlined a path for the clinical translation of RICTOR/mTORC2 inhibition in the treatment of melanoma. Moreover, the selective negative effect of disrupting the mTORC2–RAS interaction in resistant cells provides mechanistic insight into the potential requirement for mTORC2 in the resistance of melanoma to targeted therapy.

## Data Availability

Data is contained within the article or supplementary material.

## Supplementary Materials

The following are available online at https://www.mdpi.com/article/10.3390/biomedicines9101498/s1, Figure S1: Survival analysis of melanoma patients, Figure S2: RICTOR overexpression promotes cell growth in 3D but inhibits cell growth in 2D, Figure S3: RICTOR overexpression induces sensitivity to STAT3 inhibitor, Figure S4: mTOR inhibitor induces a transient reduction in AKT phosphorylation, Figure S5: Cells resistant to the BRAF inhibitor are very sensitive to mTORC2 inhibition, Figure S6: The mTORC2 inhibitor (JR-AB2-011) disrupts mTOR interaction with RICTOR and with RAS, Figure S7: RAS interacts with RICTOR in resistant melanoma. Figure S8: Quantification of the blots from Figure 2A, Figure 4A,D and Figure 6B, Table S1: Clinical data.
