# Supplementary material for: RICTOR Affects Melanoma Tumorigenesis and Its Resistance to Targeted Therapy
Source: Biomedicines. 2021 Oct 19;9(10):1498. doi: 10.3390/biomedicines9101498 (PMC8533235; doi:10.3390/biomedicines9101498)
Supplement: Supplementary file 1 [file biomedicines-09-01498-s001.zip › biomedicines-1390815-supplementary.pdf]

Figure S1

A

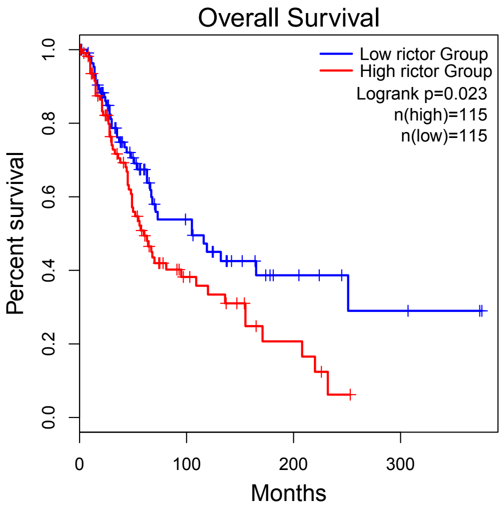

B

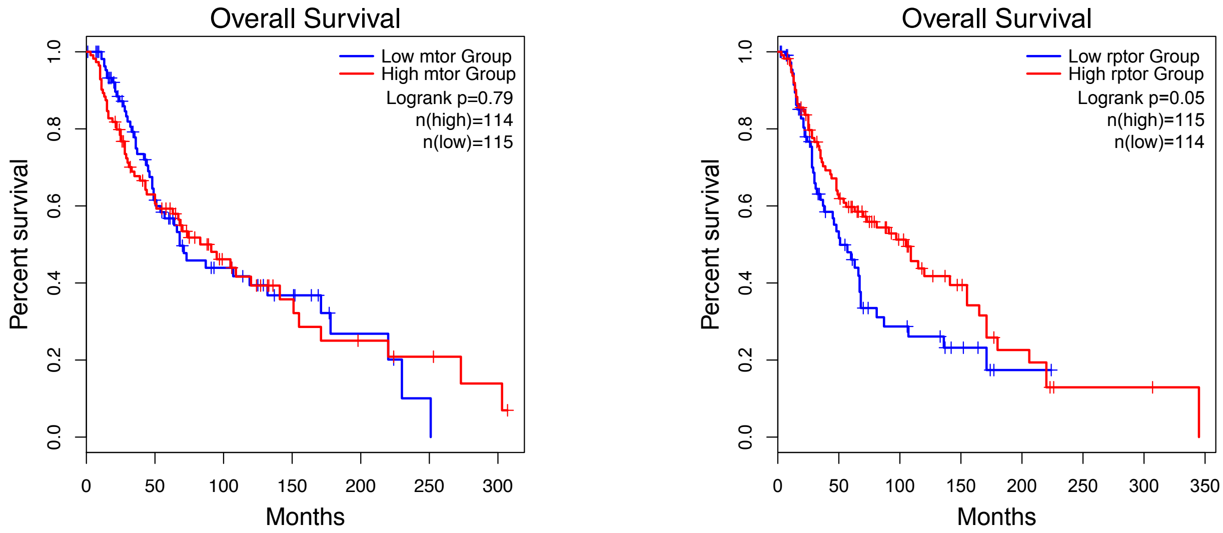

C

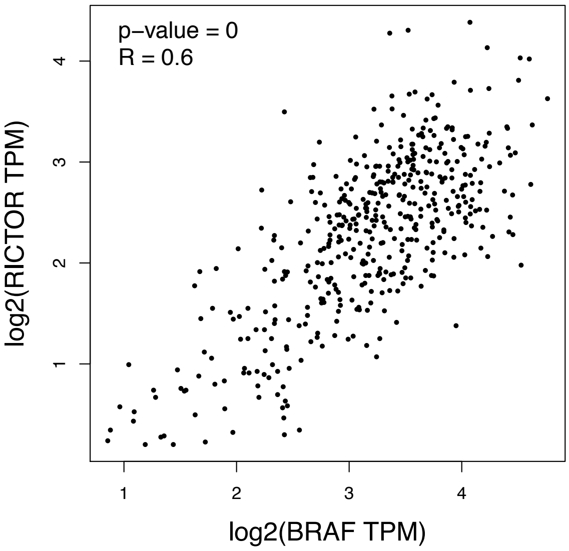

A : Survival analysis of melanoma patients (TCGA) stratified by expression of RICTOR. Cohort was split into high versus low RICTOR expression at the 75th percentile. **B :** Survival analysis of melanoma patients (TCGA) stratified by expression of mTOR (Left) or RAPTOR (Right). Cohort was split into high versus low expression at the 75th percentile. **C :** mRNA expression levels for RICTOR and BRAF in human melanoma samples, in which each dot corresponds to one tumor sample. Pearson score (R=0.6) shows a positive and significant correlation.

Figure S2

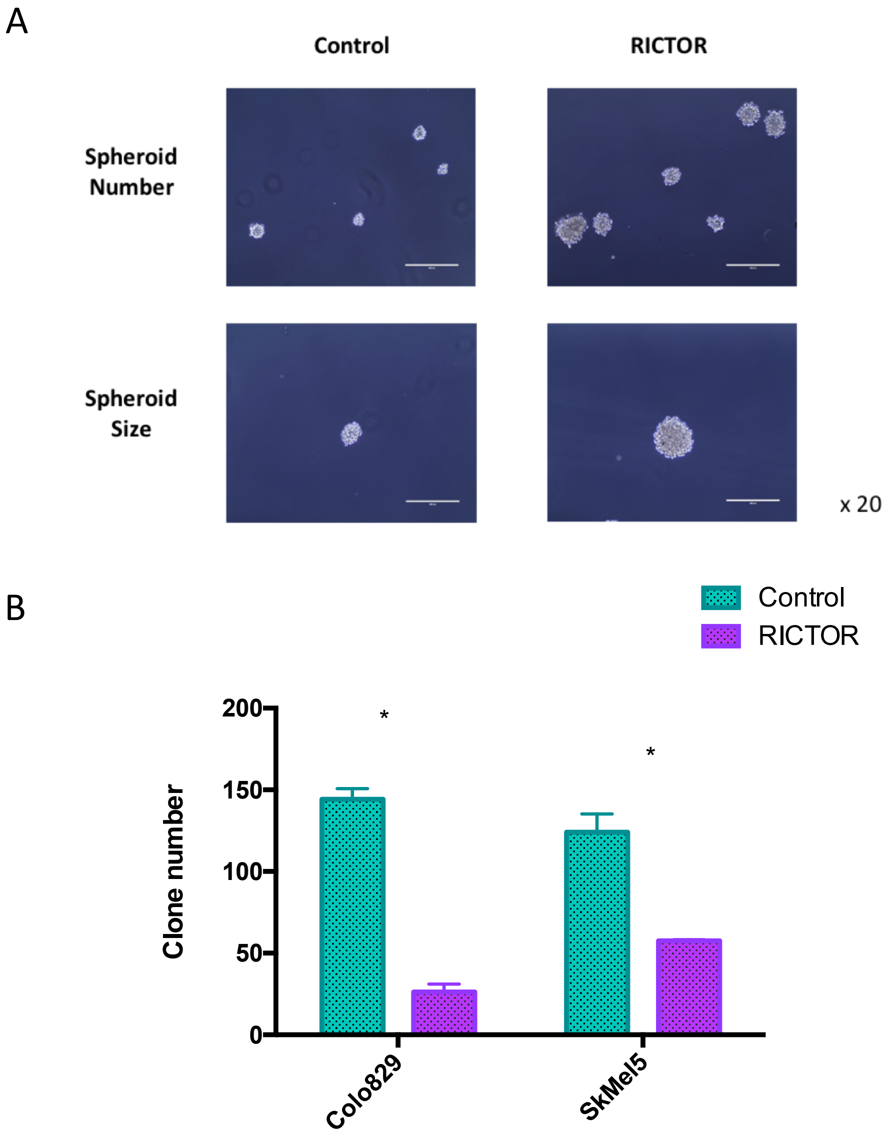

A : SkMel5 melanoma cell lines were transfected with empty vector (Control) or with vector expressing human RICTOR cDNA (RICTOR) and grown as spheroid (3D). Pictures were taken after 10 days, scale bar represents 400µm (top) and 200µm (bottom) to assess spheroid number and size.

B : Colo829 and SkMel5 melanoma cell lines were transfected with empty vector (Control) or with vector expressing human RICTOR cDNA (RICTOR) and selected by resistance to blasticidin. Colonies grown in 2D were counted after 14 days. Graphs represent mean ± s.d. of an experiment in triplicate (similar results were obtained in three independent experiments). Significance was calculated using the Student's t test: *$p < 0.05$.

Figure S3

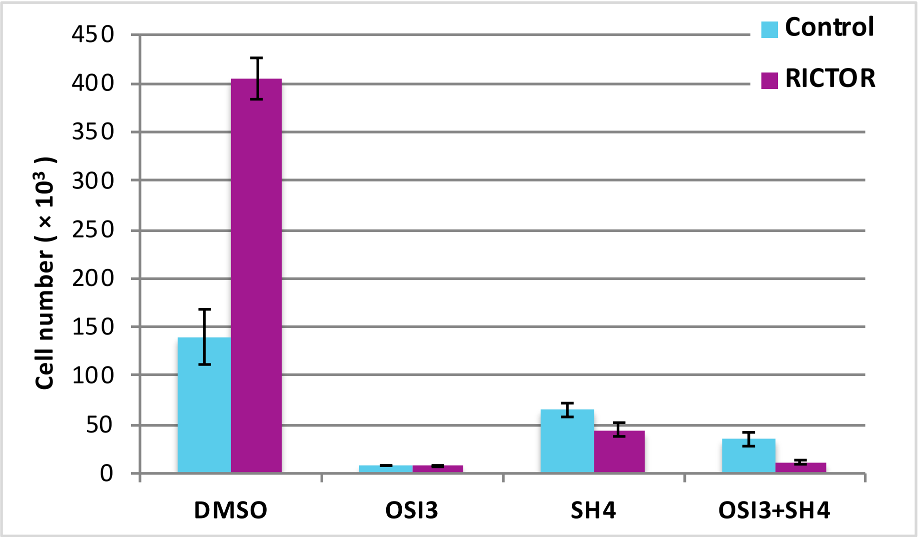

SkMel5 melanoma cell lines were transfected with empty vector (Control) or with vector expressing human RICTOR cDNA (RICTOR) grown as spheroids (3D) and treated with DMSO, OSI-027 1µM, SH4-54 2µM or both treatment combined. Cells were dissociated after 10 days of treatment and counted. Graphs represent mean ± s.d. of an experiment in triplicate.

Figure S4

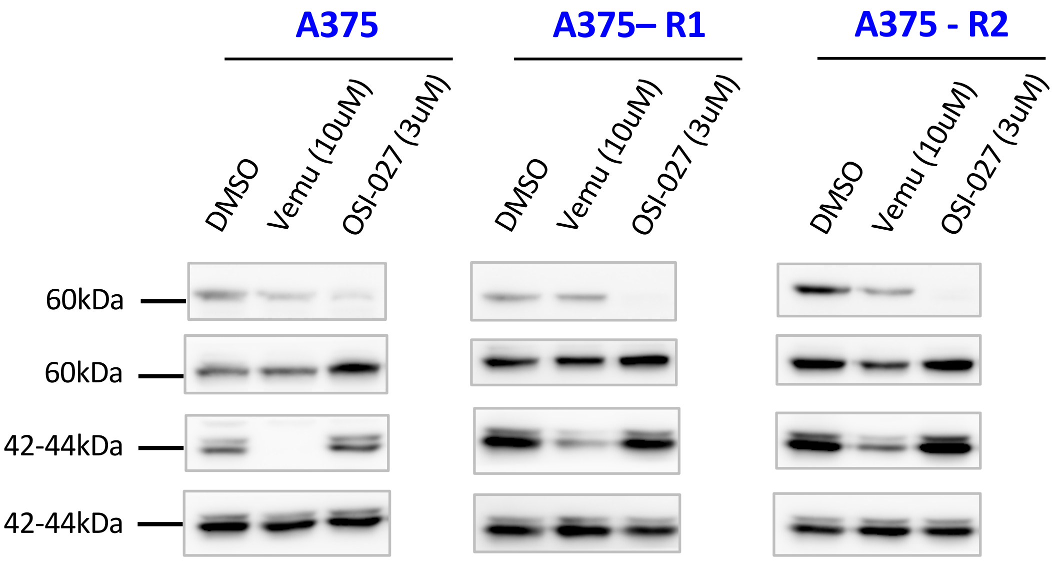

A375 melanoma cell line and A375 Vemurafenib resistant cell lines (A375-R1 and A375-R2) were treated for 1 hour with DMSO, Vemurafenib (10µM) or OSI-027 (3µM) and expression of phosphorylated AKT and ERK and total AKT and ERK were assessed by western blotting.

Figure S5

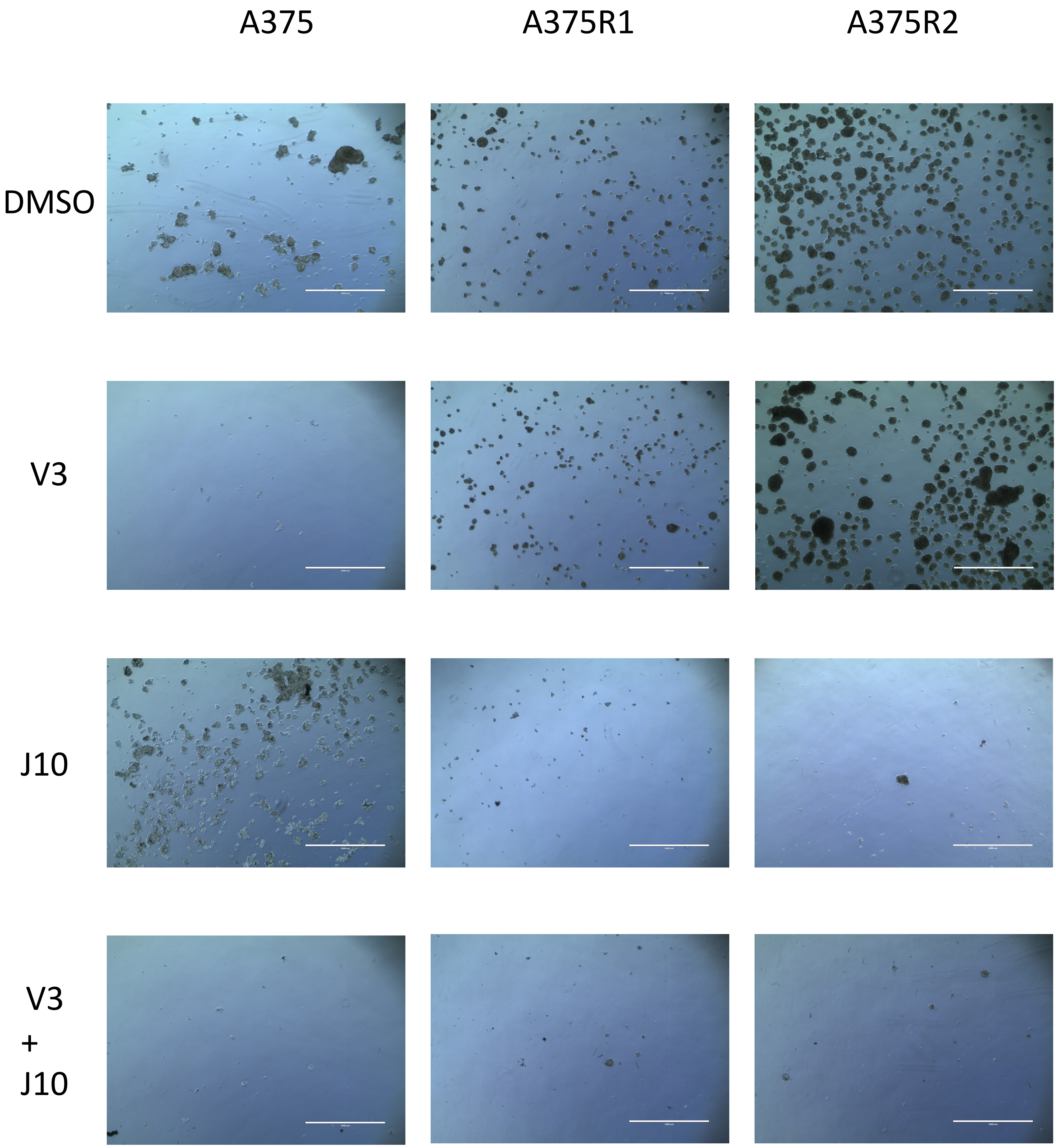

A375 parental and resistant (A375R1, A375R2) cell lines were grown as spheroids (3D) and treated with DMSO, 3µM Vemurafenib (V3), 10µM JR-AB2-011 (J10) or the combination of both (V3+J10) for 10 days. Pictures were taken after 10 days of treatment; scale bar represents 1000 µm.

Figure S6

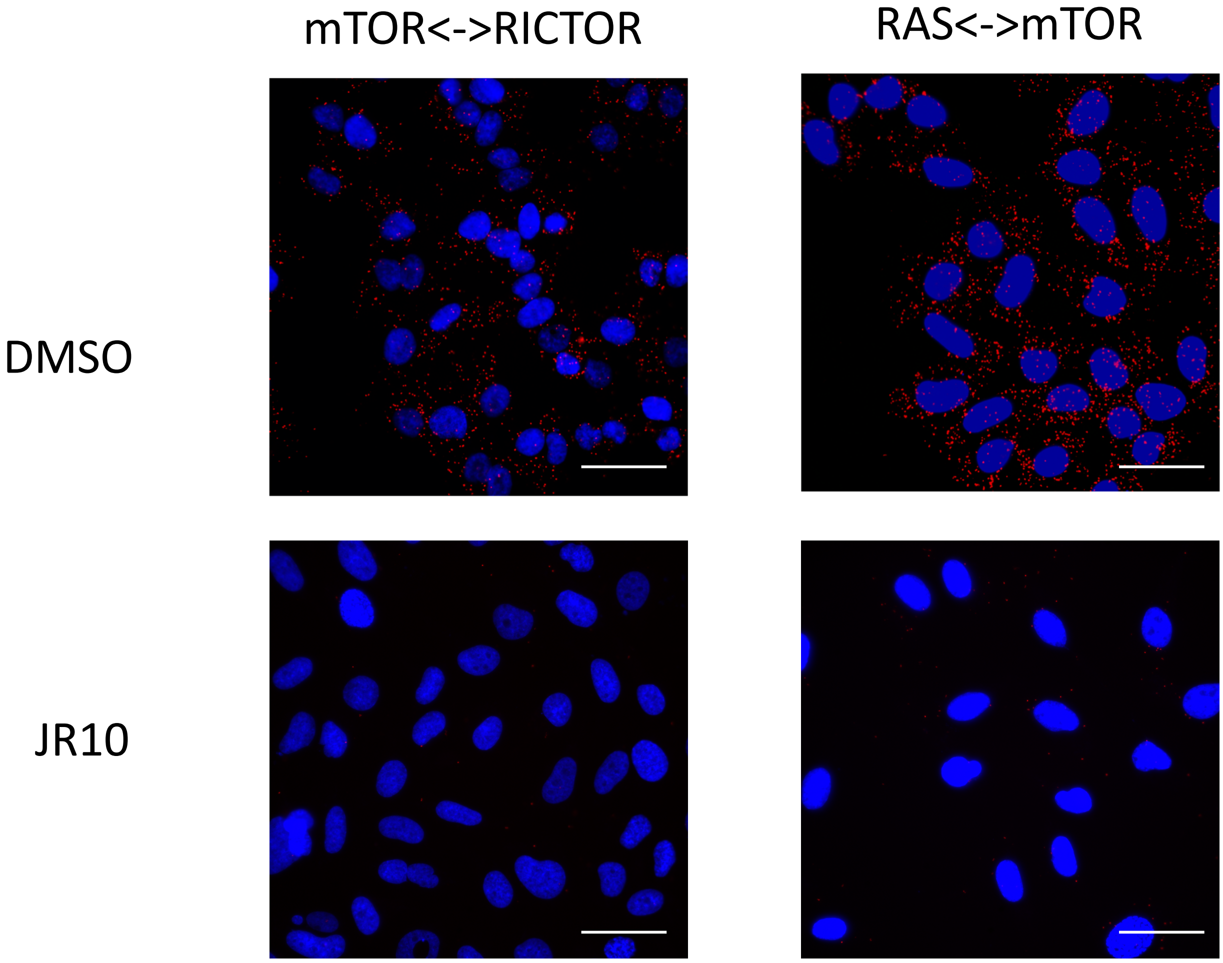

A375-R1 cells grown in 2D were treated with DMSO or 10 µM (JR10) of JR-AB2-011 for 24 hrs and RICTOR interactions with mTOR or RAS were detected by in situ PLA. The interactions were visualized as fluorescent red dots. DAPI stained nuclei (blue). Scale bar, 50 µm.

Figure S7

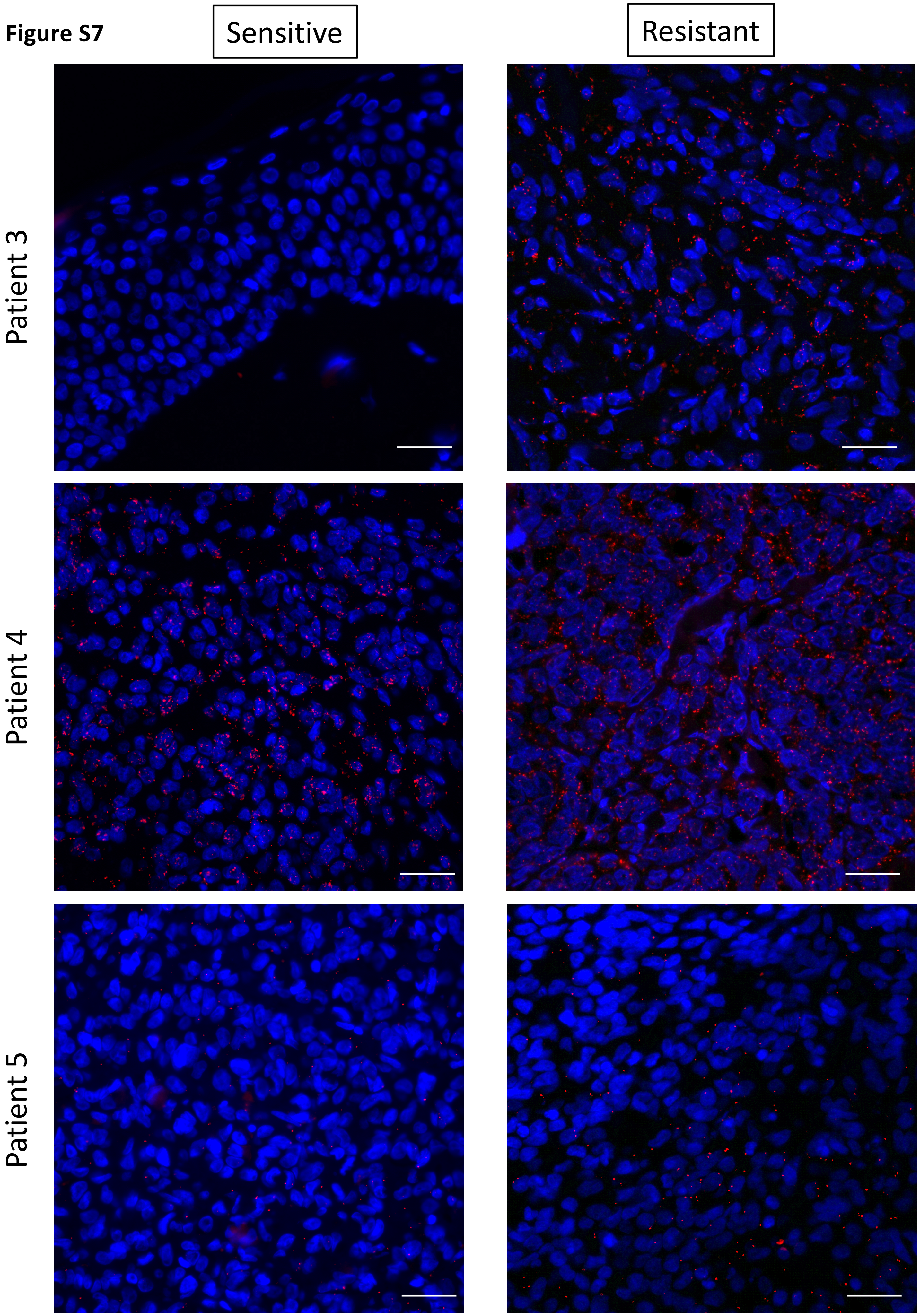

Representative pictures of RAS-RICTOR interactions assessed by in situ PLA (red dots) in paraffin sections of 3 metastatic human melanoma samples before treatment and after resistance occurred. DAPI-stained cell nuclei (blue). Scale bar, 50 µm.

Figure S8

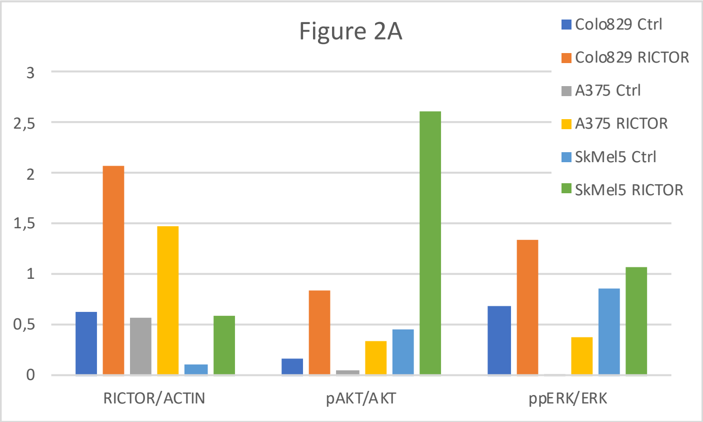

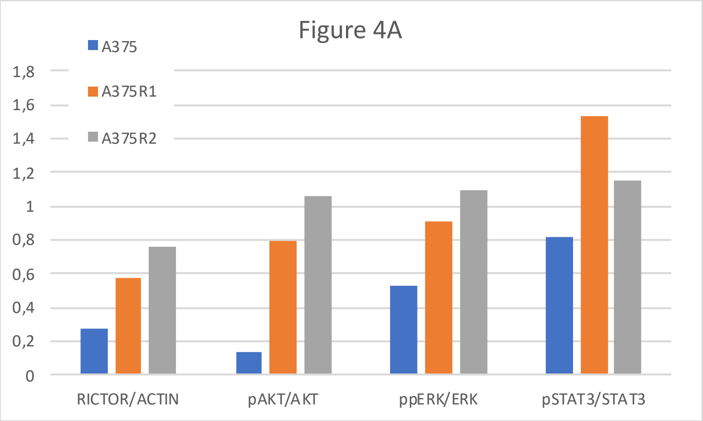

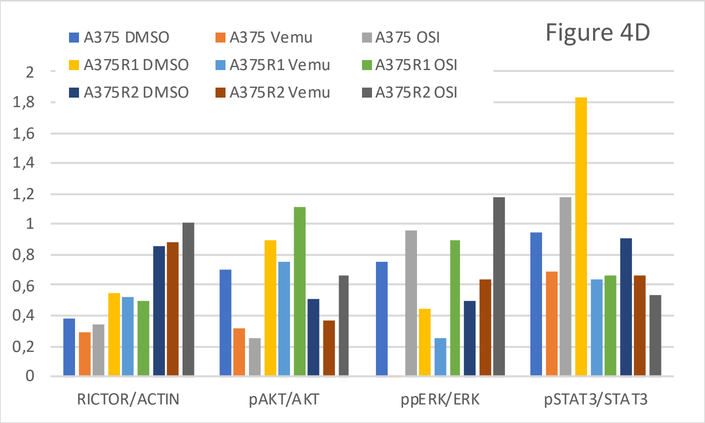

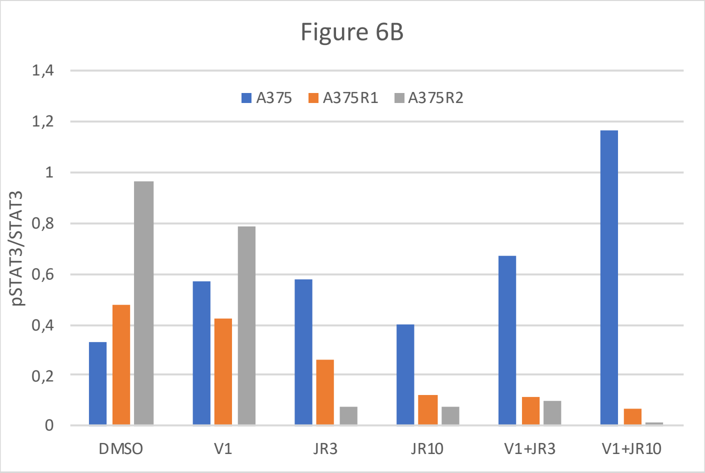

Quantification of the blots from Figures 2A, 4A, 4D and 6B

Supplementary Table S1

Patients	Firts line of Therapy			Biopsy before treatment		Biopsy at resistance	
	Inhibitors	Beginning	End	Location	Date	Location	Date
1	Dabrafenib+Trametinib	19/10/2012	21/04/2015	Right inguinal lymph node§	16/09/2011	Right iliac lymph node§	29/08/2014
2	Dabrafenib+Trametinib	20/02/2015	27/07/2015	Left ear*	20/02/2015	Left ear§	15/11/2016
3	Dabrafenib+Trametinib	11/07/2016	11/10/2017	Right breast§	24/05/2016	Thorax§	11/10/2017
4	Dabrafenib+Trametinib	12/04/2019	16/08/2019	Left iliac lymph node§	12/04/2019	Liver§	16/08/2019
5	Dabrafenib+Trametinib	24/11/2017	29/05/2018	Right inguinal lymph node§	17/11/2017	Right iliac lymph node§	15/05/2018

*primary tumor ; §Metastatic tumor
